# Sympathetic limitation of exercise hyperemia: even hypoperfused muscle is not exempted

**DOI:** 10.3389/fphys.2012.00411

**Published:** 2012-10-23

**Authors:** D. J. Duncker, I. H. Heinonen

**Affiliations:** ^1^Division of Experimental Cardiology, Thoraxcenter, Erasmus MC, University Medical Center RotterdamRotterdam, Netherlands; ^2^Research Centre of Applied and Preventive Cardiovascular Medicine, University of Turku and Turku University HospitalTurku, Finland; ^3^Turku PET Centre, University of Turku and Turku University HospitalTurku, Finland

Exercise requires major adjustments in cardiovascular performance to accommodate the large increases in blood flow to active skeletal muscle groups. These cardiovascular adjustments involve alterations in autonomic control, most notably an increase in sympathetic activity, that act not only to increase cardiac output but also redistribute blood flow away from visceral organs and inactive skeletal muscle to enable a sufficient increase in flow to the active muscle while maintaining aortic perfusion pressure (Laughlin et al., 2012). Sympatholysis in active skeletal muscle facilitates the increase in flow that occurs during exercise, but there is evidence that even during severe exercise sympathetic vasoconstriction limits blood flow in the active skeletal muscle (Laughlin et al., 2012). Under normal inflow conditions this sympathetic restraint of flow has minimal effects on muscle oxygenation, as the limitation of flow can be compensated for by an increase in muscle oxygen extraction (Laughlin et al., 2012). In contrast, in the presence of a flow-limiting artery stenosis the sympathetic vasoconstrictor influence may interfere with autoregulation of muscle blood flow, thereby aggravating tissue hypoperfusion. There is evidence that intense sympathetic activity can limit ischemic vasodilation of skeletal muscle resistance vessels, as sympathetic activation was shown to limit reactive hyperemia in the human fore-arm (Ardill et al., 1967). However, the effect of a flow-limiting stenosis on sympathetic control of skeletal muscle flow during exercise remains to be established.

In a series of studies published over the past 5 years, Casey and Joyner and colleagues have explored the cardiovascular adjustments in response to skeletal muscle hypoperfusion during exercise (Casey and Joyner, 2011). For this purpose they developed an elegant model of forearm blood flow limitation in humans by use of an inflatable balloon positioned in the brachial artery. Using this model they have shown that inflation of the balloon during exercise results in an immediate decrease in blood flow followed by a partial (~80% of normal) restoration of blood flow. The latter is, at least in part, dependent on nitric oxide and adenosine (Casey and Joyner, 2011). In this special issue of *Frontiers in Physiology*, Casey and Joyner addressed the important question whether the incomplete restoration of blood flow distal to an acute stenosis during exercise could be the result of sympathetic activity (Casey and Joyner, 2012). The results demonstrate that the incomplete restoration of blood flow was indeed the result of a sympathetic vasoconstrictor influence in the forearm microcirculation, as the non-selective α-adrenoceptor antagonist phentolamine facilitated blood flow recovery to pre-inflation levels. These findings imply that, even during exercise in conjunction with a flow-limiting stenosis, sympathetic activity continues to limit muscle perfusion. Another interesting observation was that the level of flow recovery in an individual was inversely correlated with the vasoconstriction produced by tyramine-induced release of endogenous norepinephrine, suggesting that significant inter-individual variability in sensitivity to sympathetic activation exists. These are important observations because it suggests, for the first time, that the limited exercise capacity observed in patients with obstructive peripheral artery disease (Stewart et al., 2002) is not only due to the presence of a proximal artery stenosis but also due to incomplete vasodilation of the skeletal muscle microcirculation, as a consequence of competition between α-adrenergic vasoconstriction and autoregulation. The observations are in good agreement with observations in the canine coronary circulation where α-adrenergic constriction limits resistance vessel dilation distal to a flow-limiting stenosis in dogs during treadmill exercise, thereby aggravating cardiac muscle hypoperfusion (Laxson et al., 1989).

As is usually the case with interesting data, several questions arise from the present study, which should be the subject of future studies. First, it would be important to determine whether similar results are obtained with (1) exercise involving a larger number of muscle groups, e.g., leg exercise or even whole body exercise, (2) higher intensities of exercise, and (3) more severe degrees of stenosis. Second, it would be important to repeat studies in the presence of β-adrenergic receptor blockade. Thus, it is possible that the phentolamine-induced improvement in flow restoration was, at least in part, due to presynaptic α_2_-adrenergic blockade resulting in enhanced norepinephrine release and subsequent (unopposed) vascular β_1_- and β_2_-adrenergic receptor stimulation (Laughlin et al., 2012). Third, the α-adrenergic receptor subtype(s) mediating the sympathetic vasoconstriction was not determined. It is well known that α_1_- and α_2_-receptors are differentially expressed in small arteries (α_1_) vs. arterioles (α_2_) (Faber, 1988), partly as a result of which α_2_-receptors are more sensitive to metabolic inhibition (Anderson and Faber, 1991; Wray et al., 2004). It is thus possible that α_1_-adrenergic receptors mediated the sympathetic restraint of flow. Fourth, studies should also be performed with other techniques (e.g., PET or MRI) allowing investigation of the regional blood flow responses separately in various tissues of the forearm or leg, in order to establish whether the α-adrenergic constriction actually occurred in the active skeletal muscle or that its effects on whole forearm blood flow were principally due to constriction in the inactive muscle fibers and non-muscular tissues such as bone, fat, and skin (Heinonen et al., 2012). Finally, it would be of significant interest to determine whether these findings apply to patients with peripheral artery disease, who oftentimes suffer from vascular endothelial dysfunction due to reduced nitric oxide bioavailability (Stewart et al., 2002). The authors have actually already shown in healthy human subjects that restoration of flow during hypoperfusion is critically dependent on nitric oxide (Casey and Joyner, 2009). It is likely that sympathetic limitation of flow during exercise would be even more pronounced in patients with peripheral artery disease, as lack of nitric oxide may leave the microcirculation more vulnerable to sympathetic vasoconstriction (Dinenno and Joyner, 2006; Joyner and Green, 2009). Notwithstanding these considerations, the study Casey and Joyner in this issue of *Frontiers in Physiology* provides further evidence for the flow restraining influence of sympathetic activity that is apparently strong enough to limit metabolic vasodilation in the microcirculation of exercising human skeletal muscle even in the presence of hypoperfusion.
